# A Method for Application of Classification Tree Models to Map Aquatic Vegetation Using Remotely Sensed Images from Different Sensors and Dates

**DOI:** 10.3390/s120912437

**Published:** 2012-09-12

**Authors:** Hao Jiang, Dehua Zhao, Ying Cai, Shuqing An

**Affiliations:** 1 Department of Biological Science and Technology, Nanjing University, 22 Hankou Rd., Nanjing 210093, China; E-Mails: jiang20003hao@163.com (H.J.); caiying79@126.com (Y.C.); anshq@nju.edu.cn (S.A.); 2 HydroChina Huadong Engineering Corporation, 22 Chaowang Rd., Hangzhou 310014, China

**Keywords:** aquatic vegetation, remote sensing, classification tree, sensor systems

## Abstract

In previous attempts to identify aquatic vegetation from remotely-sensed images using classification trees (CT), the images used to apply CT models to different times or locations necessarily originated from the same satellite sensor as that from which the original images used in model development came, greatly limiting the application of CT. We have developed an effective normalization method to improve the robustness of CT models when applied to images originating from different sensors and dates. A total of 965 ground-truth samples of aquatic vegetation types were obtained in 2009 and 2010 in Taihu Lake, China. Using relevant spectral indices (SI) as classifiers, we manually developed a stable CT model structure and then applied a standard CT algorithm to obtain quantitative (optimal) thresholds from 2009 ground-truth data and images from Landsat7-ETM+, HJ-1B-CCD, Landsat5-TM and ALOS-AVNIR-2 sensors. Optimal CT thresholds produced average classification accuracies of 78.1%, 84.7% and 74.0% for emergent vegetation, floating-leaf vegetation and submerged vegetation, respectively. However, the optimal CT thresholds for different sensor images differed from each other, with an average relative variation (RV) of 6.40%. We developed and evaluated three new approaches to normalizing the images. The best-performing method (Method of 0.1% index scaling) normalized the SI images using tailored percentages of extreme pixel values. Using the images normalized by Method of 0.1% index scaling, CT models for a particular sensor in which thresholds were replaced by those from the models developed for images originating from other sensors provided average classification accuracies of 76.0%, 82.8% and 68.9% for emergent vegetation, floating-leaf vegetation and submerged vegetation, respectively. Applying the CT models developed for normalized 2009 images to 2010 images resulted in high classification (78.0%–93.3%) and overall (92.0%–93.1%) accuracies. Our results suggest that Method of 0.1% index scaling provides a feasible way to apply CT models directly to images from sensors or time periods that differ from those of the images used to develop the original models.

## 1. Introduction

Shallow freshwater lakes are some of the ecosystems most vulnerable to anthropogenic disturbance [1,2]. With the development of socio-economic uses, global water pollution is becoming increasingly serious; consequently, more and more aquatic vegetative habitats are lost, which results directly in changes to aquatic vegetative productivity, distribution and biodiversity [3,4]. Because of the important ecological and socio-economic functions of aquatic vegetation [5,6], dynamic monitoring at large spatial scales is important for lake management. To be effective and cost-efficient, such monitoring efforts require the development of aquatic vegetation maps using remotely sensed information [7–11].

However, mostly due to the low spectral signal for aquatic vegetation in remotely sensed images, aquatic vegetation is not as easily detectable as terrestrial vegetation in these images [8,12]. Although many successful classifications of aquatic vegetation have been achieved, with accuracies ranging from 67.1 to 96% [12–18], remote sensing techniques have not been used widely as a regular tool for monitoring aquatic vegetation changes, and more research is needed to help clarify the most appropriate and effective methods [1,12,19–21].

Many remote sensing techniques have been developed to identify aquatic vegetation, including unsupervised isoclustering techniques, supervised maximum likelihood classifiers, Tasseled-Cap classification and remote sensing combined with ancillary environmental data [1,13,22–24]. However, most of these methods need either manual interpretation or abundant ground truth samples. A standard, less subjective method that is effective when ground truth samples are insufficient to evaluate the classification results is lacking. Classification trees (CT) have the potential to satisfy this need and have been used successfully [16,18,25–28]. However, in most previous studies the images used to create the CT models and those used to apply the CT model to other times or locations were generally from the same satellite sensors [16,18,26,29]. Mostly due to the differences in both band wavelengths and spectral response curves among satellite sensors, the spectral reflectance and spectral index (SI) values at the same time for the same target might be very different in different images [30,31]. This explains the difficulty associated with directly applying a CT model developed using images from a specific sensor to images from a different sensor, especially for the classification of aquatic vegetation with inherently low spectral signals [8]. Therefore, the application of CT models may be greatly restricted in many situations, such as when it is difficult to collect sufficient images from the same sensors due to cloud cover (which is a common occurrence in rainy areas such as Taihu Lake, especially during the growth periods of aquatic vegetation) and when the objective is to map aquatic vegetation for past periods in which the satellite technology was less developed, resulting in a lack of images from the same sensor.

To address the restrictions to using CT models to map aquatic vegetation, we have developed a simple normalization method for the application of CT modeling techniques to images from different sensors for Taihu Lake, China, using field measurements and satellite images from ETM+, TM, AVNIR-2 on the Advanced Land Observing Satellite (ALOS) and CCD on the Chinese environmental satellite of HJ-1B. In our effort to map aquatic vegetation of Taihu Lake using CT models, we used images normalized with selected pixels that incorporated the characteristics of the application image instead of the original remotely sensed images. We compared three different normalization methods to determine which gave the most consistent classification results across images.

Our approach was based on several assumptions: (1) images from different sensors with similar but different bands contain the same information regarding aquatic vegetation or related environmental factors, and thus the spectral indices that are based on them are comparable by inter-calibration [7,8]; (2) due to the differences in band wavelength ranges, spectral response curves, weather conditions and other factors, the thresholds that delineate aquatic vegetation from other types in CT models developed using images from different sensors must be different from each other; and (3) by selecting typical and stable pixels characteristic of the application image as the standards by which to normalize the images, we can apply the CT model developed from the normalized images directly to images from different sensors to successfully delineate aquatic vegetation types.

## 2. Materials and Methods

### 2.1. Site Description

Our study area consisted of Taihu Lake, the third-largest freshwater lake in China, where aquatic vegetation distribution has been experiencing a significant change during the past decades [32–34], as well as the surrounding area within 500 m of the lake boundary, where most of the reed vegetation (one of the most common types of emergent vegetation) was distributed. Taihu Lake is located in the core of the Yangtze Delta, one of the most developed areas in China, within the lower reaches of the Yangtze River Basin [35]. The lake, with an average depth of 1.9 m, occupies a surface area of 2,425 km^2^. Its catchment contains 3.7% of the country's population and 11.6% of its gross domestic product (GDP) within its area of 36,900 km^2^, which accounts for only 0.4% of China's land area [36]. Since the 1950s and especially since the 1980s, human activities have increasingly stressed the lake's development. Currently, the primary water problem in Taihu Lake is eutrophication, which together with human activities, are changing and destroying the formerly healthy aquatic ecosystem of Taihu Lake [37].

During our field visits in 2009 and 2010, we classified the aquatic vegetation into three types: emergent vegetation, floating-leaf and floating vegetation (because of the dominance of floating-leaf over floating vegetation in this class, we refer to it as floating-leaf vegetation in later text although it includes both), and submerged vegetation. More than 11 species of aquatic plants were found, similar to other field surveys in recent years [32,33]. *Phragmites communis*, *Nymphoides peltatum* and *Potamogeton malaianus* dominated the emergent, floating-leaf and submerged vegetation, respectively. Because emergent vegetation has the highest signal intensity and submerged vegetation has the lowest, areas that consisted of emergent vegetation mixed with other aquatic vegetation types were classified as emergent vegetation, and areas with mixed floating-leaf and submerged vegetation were classified as floating-leaf vegetation.

### 2.2. Field Surveys

We conducted field surveys on 14–15 September 2009 and 27 September 2010. In 2009, a total of 426 training or validation samples were obtained from: (a) 208 plots located along a transect from the east to the south of the lake; (b) 137 plots from 26 lake locations distributed nearly uniformly across the lake [36]; and (c) 48 plots of reed vegetation and 33 plots of terrestrial land cover (e.g., shoreline roads and buildings such as docks, businesses and factories) selected from a 1:50,000 land use and land cover map. Similarly, a total of 539 field samples were obtained in 2010, including 438 photographs taken along a transect from the east to the southeast of the lake and 101 plots from the 1:50,000 land use and land cover map. The field survey has been described in detail by Zhao *et al.* [29].

### 2.3. Image Processing

Because they contain dynamic information concerning aquatic vegetation and related environmental factors, multi-seasonal images have the potential to provide higher classification accuracy than a single image [16,38]. Therefore, in this study we used a combination of two images for aquatic vegetation identification, one from winter and one from summer. A total of six image pairs were used: (1) ETM+ images dated 26 March and 17 August 2009 (SLC-off images downloaded from http://earthexplorer.usgs.gov/); (2) TM images dated 13 January and 10 September 2009; (3) AVNIR-2 images from ALOS dated 30 December 2008 and 17 August 2009; (4) CCD images from HJ-1B dated 15 March and 10 September 2009; (5) ETM+ images dated 13 March and 21 September 2010; and (6) CCD images from HJ-1B dated 10 March and 21 September 2010. Of these image pairs, the four from 2009 (including the AVNIR-2 image dated 30 December 2008 because no high quality AVNIR-2 image could be obtained from the winter of 2009) were used to compare different normalization methods, while the other two pairs were used to validate the robustness of our recommended normalization method. The band wavelength ranges and resolutions of the images used in this study are shown in Table 1. For all the images used, cloud cover was no greater than 10% of the entire study area, following the recommended standard for aquatic remote sensing [39]. Before atmospheric correction, cloud-contaminated pixels were removed using interactive interpretation. The cosine approximation model (COST) described by Chavez, which has been used successfully in other aquatic remote sensing studies for atmospheric corrections of multi-temporal Landsat images [40,41], was used for the atmospheric corrections, and thus surface reflectance images were obtained for the calculation of spectral indices.

In order to reduce misclassification resulting from the presence of algae, cyanobacterial blooms must be detected before delineation of the aquatic vegetation classes. The method used here was similar to the procedure by Zhao *et al.* and is briefly summarized here. We first divided Taihu Lake into two parts: the eastern and southeastern portions, referred to as the grass type zone, and the remaining portions, referred to as the algae type zone [42]. The grass zone consisted of large areas of floating-leaf and submerged vegetation where the water clarity was significantly higher than the algae type zone, and nearly no cyanobacterial blooms were found. Conversely, almost no floating-leaf and submerged vegetation was found in the algae type zone. Because there is a high probability that a pixel with NDVI > 0.4 is vegetation, we next identified the pixels in the algae type zone where NDVI > 0.4, using the ETM+ image of 20 August 2010, which was taken soon after a rainfall event caused a near absence of cyanobacterial blooms on the water surface. We then removed from the images all the pixels in the algae type zone with a higher TM4 than TM3 reflectance [43], except where the pixels had NDVI > 0.4 in the ETM+ image of 20 August 2010. Pixels that were removed were not used in the subsequent efforts to delineate and identify aquatic vegetation. In the grass type zone, if a pixel had TM4 > TM3 at least twice between August and September, it was identified as potential aquatic vegetation and retained; if a pixel had TM4 > TM3 only once during that period, it was identified as a cyanobacterial bloom area and was removed. Besides the remote sensing images discussed previously, two additional ETM+ images (dated 2 September 2009 and 19 July 2010) were used to aid in detecting cyanobacterial blooms. Using ERDAS IMAGINE 9.2 (Leica Geosystems Geospatial Imaging, LLC), geometric corrections were applied to all the images using second-order polynomials with an accuracy higher than 0.5 pixel.

### 2.4. Analytical Methods

#### 2.4.1. Classification Tree Model Structure

Classification tree (CT) analysis, which partitions data dichotomously using thresholds determined from application of specified splitting rules, was used for the identification and mapping of aquatic vegetation. Considering the differences in both wavelength range and the spectral response curve among images from different sensors, we first developed CT model structures manually for emergent, floating-leaf and submerged vegetation based on known relationships between spectral indices and the aquatic vegetation in Taihu Lake, and, secondly, we obtained quantitative thresholds for specified remotely sensed images from CT analysis of the data in our statistical software package (PASW-Statistics v. 18) using the Chi-squared Automatic Interaction Detector (CHAID) algorithm. In this paper, the thresholds obtained from the quantitative analysis were considered optimal thresholds based on field observations. This process differed from other studies in which the CT models, both structure and thresholds, were developed directly from specified images and ground measurements using quantitative algorithms [16,18,25,26]. Our process was based on the consideration that, if we obtained both structure and thresholds using a quantitative algorithm, different CT structures would be obtained for different images, and thus it would be difficult to compare the optimal thresholds for the images from different time periods and sensors and to evaluate the influence of different image time periods and sensors on the optimal thresholds of the CT model. To develop model structures, we selected three of the most sensitive and stable spectral indices for identification of emergent, floating-leaf and submerged vegetation: the Normalized Difference Vegetation Index (NDVI) [44], Normalized Difference Water Index of McFeeters (NDWIF) [45], and average reflectance of the blue, green and red bands from the remote sensing image (AVE123) [46]. The variables were calculated by season. For example, NDVI-(w), NDVI-(s) and NDVI-(s-w) are the NDVI of winter, NDVI of summer and NDVI of summer minus NDVI of winter, respectively.

**Figure 1.**
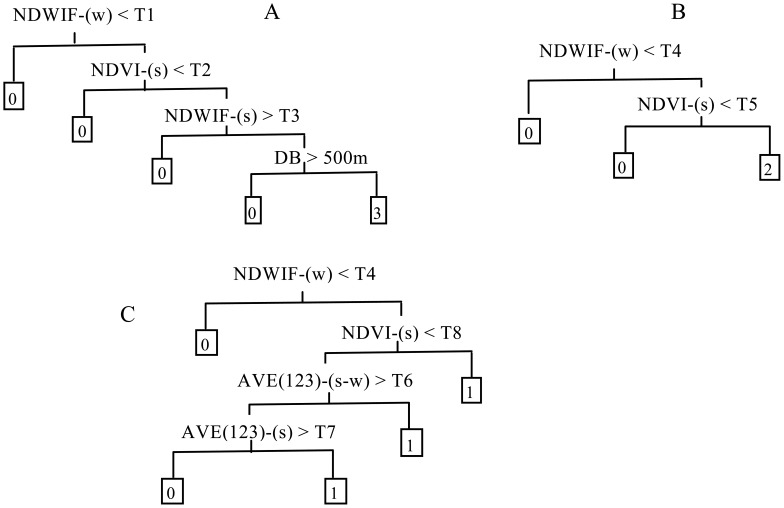
Classification tree model structure established for (**A**) emergent vegetation; (**B**) floating-leaf vegetation and (**C**) submerged vegetation. The numbers 3, 2 and 1 in the end nodes of the classification trees represent emergent, floating-leaf and submerged vegetation, respectively, whereas 0 represents other types. T1, T2, T3, T4, T5, T6, T7 and T8 represent the quantitative thresholds obtained using classification tree analysis of the field observations in PASW-Statistics v. 18. Variables used are the Normalized Difference Water Index of McFeeters (NDWIF), the Normalized Difference Vegetation Index (NDVI), the average reflectance of the blue, green and red image bands (AVE123) and the distance to the lake bank (DB). Variables were calculated by season (s = summer, w = winter) or differences among seasonal values (e.g., s-w).

Figure 1 shows the basic CT model structures we developed for identification of emergent, floating-leaf and submerged vegetation, which differed slightly from those presented by Zhao *et al.*, primarily because of the lack of a band corresponding to ETM+ band-5 in the ALOS and HJ images. The model structures here were developed in a progressive fashion (*i.e.*, first emergent, then emergent + floating-leaf and then emergent + floating-leaf + submerged vegetation), treating the remaining types as “other”. For emergent vegetation, we assigned the primary spitting rule of NDWIF-w > T1 to obtain pixels of the lake, marsh and wasteland where emergent vegetation grew and to remove pixels representing agricultural fields and developed land. Because NDVI is generally larger for emergent vegetation than for floating-leaf vegetation, the secondary splitting rule, NDVI-s > T2, was assigned to segregate pixels of emergent vegetation and portions of the floating-leaf vegetation from pixels of open water, submerged vegetation and the bulk of the floating-leaf vegetation. NDWIF < T3 was assigned as a tertiary splitting rule to further segregate pixels of floating-leaf vegetation from pixels of emergent vegetation. At this point, some pixels of floating-leaf vegetation may still have been retained in the emergent vegetation grouping (*i.e.*, the pixels with the densest floating-leaf vegetation). Therefore, we added a fourth-level spitting rule of DB < 500 m, where DB is distance to the nearest bank, based on our field observations that most of the emergent vegetation grew within 500 m of the lake bank.

In order to separate floating-leaf vegetation from other types, only two levels of splitting rules were needed (Figure 1(B)), and these splitting rules were similar to those for emergent vegetation. The initial spitting rule of NDWIF-w > T4 was assigned to segregate the pixels of the lake where floating-leaf vegetation grew from the land pixels. Compared with open water and submerged vegetation, floating-leaf vegetation has significantly different spectral characteristics, especially at near infrared (NIR) wavelengths, due to the strong absorption of water, and thus the secondary spitting rule of NDVI > T5 was assigned.

For submerged vegetation, the initial splitting rule was again assigned to be NDWIF-w > T4 to segregate the lake pixels from the land pixels (Figure 1(C)). Because submerged vegetation can only grow in water with relatively high transparency, the water clarity of most submerged vegetation pixels is higher than that of open water pixels. AVE123 is used widely to estimate water clarity [46]; therefore, we used AVE123-(s-w) and AVE-(s) in our classification model to segregate pixels of submerged vegetation from those of open water. These splitting rules were able to identify most pixels of submerged vegetation, but some pixels of submerged vegetation have similar but weaker spectral characteristics (*i.e.*, higher reflectance of the NIR band and lower reflectance of the red band), especially where the vegetation has large leaves growing laterally near the water surface. For these pixels, AVE123 values are not substantially lower than in open water pixels, so the rules associated with these values cannot be used to effectively separate out submerged vegetation. Consequently, we assigned an alternative splitting rule of NDVI-s > T8 to identify submerged vegetation pixels with these characteristics.

#### 2.4.2. Normalization Methods

One of the most important steps in determining the effectiveness of our CT procedure was to identify pixels within each remotely-sensed image that were fully characteristic of that image. To determine the best normalization procedure, we employed and compared three different methods that applied a series of pre-processing steps to the surface reflectance image:

##### Method of 5% DN scaling

We first normalized the surface reflectance image using pixels with extreme reflectance values (*i.e.*, the highest and lowest 5% in the entire image) and subsequently created images for the spectral indices (SI).

##### Method of 5% index scaling

We first created images for the spectral indices from the surface reflectance and then normalized the SI images using pixels with extreme SI values (*i.e.*, the highest and lowest 5% in the entire image). This was similar to Method of 5% DN scaling except the normalization was applied to the SI images rather than the reflectance images.

##### Method of 0.1% index scaling

Similar to Method of 5% index scaling, normalization was applied to the SI images rather than the surface reflectance images. However, instead of normalizing the images using the highest and lowest 5% of SI pixel values for all SI as in Method of 5% index scaling, the normalization used different percentages of extreme high and low values for different indices. For NDVI and NDWIF images, the normalization parameters were calculated using pixels with the highest and lowest 0.1% of values in the entire image. For the AVE123 image, the normalization parameters were calculated using the pixels with the lowest 0.1% and highest 10% of values.

#### 2.4.3. Comparison Methods

To evaluate the relative differences in the optimal thresholds of the classification trees for the four image pairs from 2009, we computed Relative Variation (RV):

(1) $$RV=\frac{\sum_{n=1}^{4}(SI-\bar{SI})}{4\times (SI_{\text{max}}-SI_{\text{min}})}$$

where SI is the optimal threshold obtained from quantitative analysis of field data for a certain image pair, $\bar{SI}$ is the average of the optimal thresholds for the four 2009 image pairs, and SI_max_ and SI_min_ are average values of the spectral index for the 5% of pixels with the highest and lowest values, respectively, in the four image pairs.

For comparison of CT models developed from SI images normalized by Method of 5% index scaling and Method of 0.1% index scaling, Equation (1) could be simplified as follows:

(2) $$RV=\frac{\sum_{n=1}^{4}(SI-\bar{SI})}{4}\times 100$$

Classification accuracy and overall accuracy were used to assess the performances of different CT models in identifying aquatic vegetation. Classification accuracy was defined as the percentage of correctly classified samples for a certain cover type relative to the total number of actual samples of this type plus the samples mistakenly classified as this type. For example, assume there are a total of 220 samples consisting of 100 submerged vegetation and 120 other type samples. Of these, 70 submerged vegetation samples and 100 other type samples are correctly classified. Therefore, the classification accuracy for submerged vegetation is 70% (*i.e.*, $\frac{70}{100+20}\times 100=58.3\%$). The overall accuracy was defined as the percentage of samples that were classified correctly (using the above numbers, overall accuracy is 77.3%, *i.e.*, $\frac{70+100}{220}\times 100=77.3\%$) [13–18]. Generally, classification accuracy was lower than overall accuracy because of the difference in calculation method.

The quantitative analyses were likely to result in differences in the optimal thresholds of the CT models for different image pairs. In other words, RV was not zero, generally. To assess the effect of variability in the thresholds, we applied the optimal thresholds for each image pair as well as the thresholds developed for the other three image pairs to the CT models for each image pair and compared the resulting classification accuracies.

Performance of each normalization method was assessed using the classification and overall accuracies of the vegetation maps resulting from the models applied to images from each method, and the method with the best performance was selected for further analyses. The optimal thresholds of the CT models from the four image pairs were averaged for the selected normalization method, and averages were used as thresholds in final CT models for 2009. To evaluate the robustness of the models, the 2009 CT models were applied to the two 2010 image pairs (ETM+ and CCD sensors) normalized by the selected normalization method.

## 3. Results

### 3.1. Traditional CT Models for Original Images

**Figure 2.**
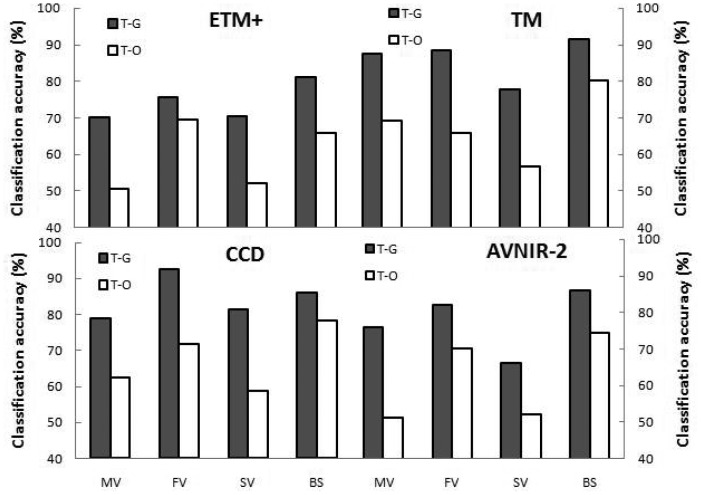
Aquatic vegetation classification accuracy (CA) of the CT models using non-normalized images. For each sensor, T-G is the CA of models with optimal thresholds developed from field data and images from that sensor, whereas T-O is the average CA of the models using thresholds developed for the image pairs from the other three sensors.

We used CT analysis with our established model structures (see Figure 1) to obtain optimal thresholds for the original image pairs from each sensor (Table 2). With the optimal thresholds, CT models obtained classification accuracies ranging from 70.1% to 87.6% for emergent vegetation, from 75.6% to 92.4% for floating-leaf vegetation, from 66.3% to 81.4% for submerged vegetation and from 81.1% to 91.6% for other types, with respective averages of 78.1%, 84.7%, 74.0% and 86.2% (Figure 2). These results suggested that aquatic vegetation types in Taihu Lake could be distinguished using pre-developed CT model structures with optimal thresholds obtained from quantitative CT analysis of field observations.

However, optimal thresholds of the models differed according to the sensor from which the image pair originated, with RV ranging from 2.50% to 13.4% (average 6.40%, Table 2). Despite relatively low values of RV, the variability in thresholds substantially affected the performance of the CT models in identifying aquatic vegetation (Figure 2). Applying thresholds developed for other image pairs (sensors) to CT models for a specific image pair (sensor) resulted in classification accuracies that averaged 59.5%, 68.0%, 62.1% and 67.8% for the images from ETM+, TM, AVNIR-2 and CCD sensors, respectively; these averages were significantly lower than the classification accuracies of CT models using the optimal thresholds for each sensor (*p* = 0.00). These results suggested that applying CT models using thresholds optimized for images from a particular sensor or date to images from different sensors or dates could reduce the classification accuracy significantly.

### 3.2. CT Models for Normalized Images

**Figure 3.**
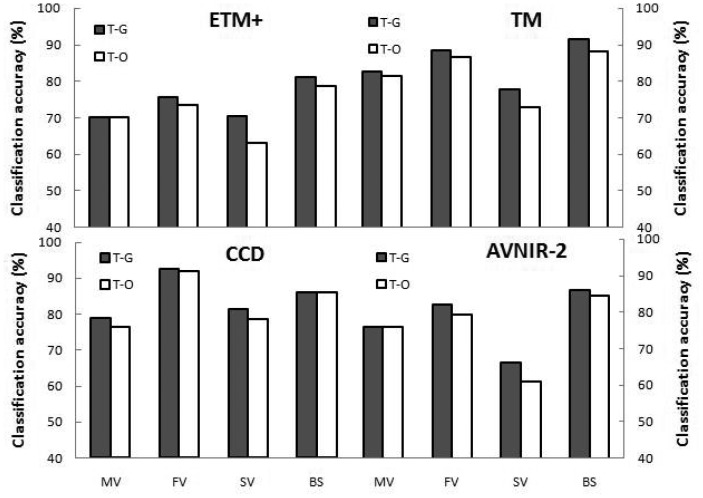
Aquatic vegetation classification accuracy (CA) of the CT models using images normalized with Method of 0.1% index scaling. For each sensor, T-G is the CA of models with optimal thresholds developed from field data and images from that sensor, whereas T-O is the average CA of the models using thresholds developed for the image pairs from the other three sensors.

Similarly, we used the field data and quantitative analysis to obtain optimal thresholds for the CT models based on the three sets of normalized images (Table 3). RV values ranged from 4.88% to 14.9% (average 9.40%) for Method of 5% DN scaling, 2.37% to 7.24% (average 5.12%) for Method of 5% index scaling and 0.28% to 3.96% (average 1.98%) for Method of 0.1% index scaling. Compared with both the RV values of the thresholds based on the original images that were not normalized and the RV values based on the normalized images for Method of 5% DN scaling and Method of 5% index scaling, the RV values for Method of 0.1% index scaling decreased significantly (*p* = 0.00). Therefore, Method of 0.1% index scaling was the best choice for resolving the problems inherent to application of CT models to images from different sensors or different dates. Classification accuracies of the CT models developed for images from a particular sensor that were normalized using Method of 0.1% index scaling (Figure 3) were very similar to those based on the original, non-normalized images (Figure 2). When applied to images from a particular sensor, the CT models developed for image pairs from other sensors achieved average classification accuracies of 76.0%, 82.8%, 68.9% and 84.4% for emergent vegetation, floating-leaf vegetation, submerged vegetation and other types, respectively, which was consistently slightly lower than that of the CT model developed specifically for that sensor. In other words, the slight variation in model thresholds among the different image pairs normalized by Method of 0.1% index scaling caused a decrease in classification accuracy. However, the magnitude of the decrease was significantly lower than that resulting from the models based on both the original images and the other normalization methods (with *p* values ranging from 0.00 to 0.01), consistent with the results associated with the RV values of the model thresholds.

### 3.3. Application of 2009 Models to 2010 Images

Applying CT models developed for normalized (Method of 0.1% index scaling) image pairs from 2009 to image pairs from 2010 resulted in high classification accuracies for models based on both the ETM+ (78.0% to 90.6%) and CCD (80.7% to 93.3%) sensors (Tables 4 and 5).

**Figure 4.**
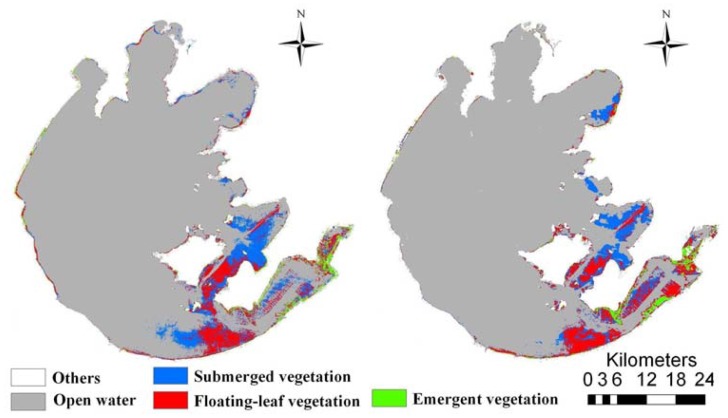
The distribution of aquatic vegetation in Taihu Lake in 2009 (**left**) and 2010 (**right**) as specified by CT models developed from 2009 images.

Overall accuracies were 92.0% and 93.1% for ETM+ and CCD images, respectively. Validation results also indicated that our normalization procedure using Method of 0.1% index scaling adequately resolved the typical problem of decreased classification accuracy when applying models developed for images from specific sensors or dates to images from other sensors or dates. Maps of the 2009 and 2010 aquatic vegetation distribution in Taihu Lake were created by applying the 2009 models to the respective year's images (Figure 4). These maps indicated that emergent, floating-leaf and submerged vegetation occupied 21.3 km^2^, 139.4 km^2^ and 148.9 km^2^ in 2009, respectively, which amounted to 0.88%, 5.75% and 6.14% of the entire lake area. In 2010, the areas of emergent, floating-leaf and submerged vegetation were 33.1 km^2^, 145.3 km^2^ and 132.3 km^2^, respectively, corresponding to 1.36%, 5.99% and 5.46% of the entire lake area.

## 4. Discussion

### 4.1. Application of CT Models to Images from Different Sensors and Dates

For original, non-normalized images, optimal thresholds for different image pairs varied by 6.40%, on average, which was probably due to the differences in the originating sensors and dates of the image pairs. First, because the characteristics of spectral bands and spectral response curves vary distinctly from sensor to sensor, vegetation indices derived from images originating from different instruments are necessarily different and not directly comparable [47]. Differences in NDVI obtained from different sensors in previous studies have ranged from 0 to 7% [30,31]. Second, additional factors such as solar and viewing angles, satellite altitude and atmospheric conditions necessarily differ among images from different sensors and dates and affect reflectance and spectral indices of images and thus comparability of reflectance and spectral indices among those images [47]. Therefore, inter-calibration of vegetation indices from different dates and sensors is necessary to improve comparability and application ability of remote sensing [30].

We found that CT models for aquatic vegetation were very sensitive to the variability in threshold values, which might be related to the low spectral signal of aquatic vegetation. Because of the strong absorption of light by water, the spectral signal of water bodies is very weak, and reflectance values are generally no more than 10% [8]; these issues add to the difficulties involved in aquatic vegetation classification and amplify the necessity for inter-calibration of indices derived from images originating from different dates and sensors, especially for submerged vegetation. More importantly, the existence of numerous mixed pixels of open water and the various aquatic vegetation types in our study further blurred the differences in reflectance and spectral indices, resulting in a strong sensitivity of the classification output to slight variations in the thresholds of the CT models, even when the spectral characteristics differed substantially between open water and the aquatic vegetation types.

### 4.2. Differences in Performance of the Normalization Methods

An appropriate normalization method for improving the application of CT models among images from different sensors and dates should have at least two characteristics: (1) the range of minimum and maximum values used to normalize an image is wide enough to encompass the potential differences caused by factors that have a nearly uniform effect on an image and that may vary among images, such as atmospheric conditions (e.g., concentrations of carbon dioxide, ozone and water vapor), satellite altitudes, sensor features and vegetation growth periods; and (2) the normalization parameters for an image are insensitive to factors that are unevenly distributed in the image, such as thin haze stacks and turbid waters caused by localized or transient factors. These standards differ from those suggested by previous researchers [48], who have suggested that the normalization parameters should be based on man-made objects whose reflectance was independent of seasonal or biological cycles. The main reason for the different approach in our study is related to the objectives. We specifically sought to eliminate, using normalization, the slight differences among images that were caused by vegetation growth period, but this was not a focus of the other numerous relative radiometric normalization methods such as the statistical adjustments approach [49,50] and histogram matching integrated in some image processing software packages.

Model performance was lowest using Method of 5% DN scaling for normalization, suggesting that the reflectance images were not especially useful as normalization objects to improve the ability of a CT model developed for a particular image to correctly classify aquatic vegetation types using images from different sensors and dates. The poor performance of Method of 5% DN scaling might be related to the effect of unevenly distributed factors (e.g., cloud stacks) on the maximum and minimum reflectance values used as normalization parameters. In other words, pixels with extreme reflectance values may have been located primarily in areas contaminated by clouds, cloud shadows or localized turbid waters. Although the pixels that were strongly contaminated by clouds were removed using interactive interpretation, less dense cloud cover remained in the images, leading to uncertainty in the normalization parameters as well as the spectral indices calculated from the normalized reflectance images. In fact, Method of 5% DN scaling is very similar in basic principle to the atmospheric correction model (COST) that we applied to the images [40,41]. Therefore, that analysis can also help explain why RV for the original images in Table 2 was relatively high.

Method of 5% index scaling was based on the normalized spectral index (SI) images. Because spectral indices generally have a better ability to resist the effect of transient or localized factors than reflectance, the pixels with extreme SI values generally are not located in areas that are drastically contaminated by these transient or localized factors. Therefore, Method of 5% index scaling was able to partially compensate for the limitations of Method of 5% DN scaling and thus exhibited better performance. To clarify further using the individual spectral indices, pixels with the maximum values of NDWIF were generally located in areas dominated by submerged vegetation, and minimum NDWIF values were typical of emergent and floating-leaf vegetation [16]. Maximum NDVI values were generally found where emergent and floating-leaf vegetation was dominant, and minimum NDVI was typical of submerged vegetation. In other words, the pixels selected by Method of 5% index scaling to calculate normalization parameters typically were not located in areas that were strongly influenced by transient or localized factors such as cloud stacks. As a result, the pixels selected by Method of 5% index scaling to calculate the normalization parameters better characterized the image than did those selected by Method of 5% DN scaling. The optimal CT thresholds for NDWIF and NDVI obtained using Method of 5% index scaling were consistently less variable (*i.e.*, lower RV) than those obtained using Method of 5% DN scaling. AVE123, the average value of the three reflectance bands, was more stable than reflectance of one band. Consequently, the CT thresholds for AVE123 obtained using Method of 5% index scaling performed slightly better than those obtained using Method of 5% DN scaling.

Method of 0.1% index scaling, which was developed to compensate for the limitations of Method of 5% index scaling, performed the best and is our recommendation for an effective normalization procedure. For NDWIF and NDVI images, normalization parameters were calculated using the most extreme 0.1%, instead of 5%, of pixel values. As mentioned previously, the pixels with the most extreme NDWIF and NDVI values were located primarily in areas of active growth of aquatic vegetation. However, aquatic vegetation in Taihu Lake was distributed across only 15.8–20.9% of the area of the entire lake, and emergent vegetation encompassed no more than 3.5% [37]. Using the highest and lowest 5% of values, the composition of the selected pixels was complex and often consisted of several different types, resulting in unstable average values of the spectral indices of these pixels. Conversely, using the highest and lowest 0.1% of values as in Method of 0.1% index scaling, the selected pixels consisted mostly of the most typical aquatic vegetation type and generally were those with the highest coverage or biomass. The pixels selected using Method of 0.1% index scaling tended to be more stable and resistant to transient and localized factors that affected reflectance. Similarly, thresholds of AVE123 normalized using Method of 0.1% index scaling performed better than those normalized using Method of 5% index scaling, as measured by decreased RV values.

## Figures and Tables

**Table 1.** Sensors, spatial resolutions, and band wavelengths of the remotely sensed images used in this paper.

**Sensor**	**Resolution**	**B1**	**B2**	**B3**	**B4**
**Landsat7-ETM+**	30	0.45–0.52	0.52–0.60	0.63–0.69	0.76–0.96
**HJ-1B-CCD**	30	0.43–0.52	0.52–0.60	0.63–0.69	0.76–0.90
**Landsat5-TM**	30	0.45–0.52	0.52–0.60	0.63–0.69	0.76–0.90
**ALOS-AVNIR-2**	10	0.42–0.50	0.52–0.60	0.61–0.69	0.76–0.89

**Table 2.** Optimal thresholds for the classification tree models (see Figure 2) developed for original, non-normalized images from different sensors using 2009 field observations and quantitative CT analysis. RV is the relative variation (%) in the values of a particular threshold.

**Sensor**	**T1**	**T2**	**T3**	**T4**	**T5**	**T6**	**T7**	**T8**
**Landsat7-ETM+**	−0.238	0.508	−0.457	0.145	0.102	−0.023	0.074	0.012
**HJ-1B-CCD**	−0.109	0.411	−0.387	0.058	0.181	0.003	0.069	0.143
**Landsat5-TM**	−0.301	0.481	−0.471	−0.092	0.196	−0.034	0.063	0.148
**ALOS-AVNIR-2**	−0.135	0.525	−0.410	0.071	0.045	−0.027	0.092	0.003
**RV (%)**	7.42	2.65	2.50	6.92	4.32	13.4	9.83	5.19

**Table 3.** Optimal thresholds for the classification tree models (see Figure 2) developed for normalized images from different sensors using field observations and quantitative CT analysis. Results are shown for the three normalization methods presented in this paper. RV is the relative variation (%) in the values of a particular threshold.

**Method**	**Sensor**	**T1**	**T2**	**T3**	**T4**	**T5**	**T6**	**T7**	**T8**
Method of 5% DN scaling	**Landsat7-ETM+**	−0.225	0.656	−0.588	0.338	0.144	−0.507	0.438	0.019
	**HJ-1B-CCD**	0.011	0.794	−0.826	0.218	0.300	−0.713	0.341	0.219
	**Landsat5-TM**	−0.138	0.681	−0.275	0.265	0.169	−0.619	0.393	0.038
	**ALOS-AVNIR-2**	−0.481	0.738	−0.631	−0.206	0.044	−0.546	0.414	−0.125
	**RV (%)**	14.6	4.88	14.9	14.8	7.03	6.98	2.94	9.08
Method of 5% index scaling	**Landsat7-ETM+**	−0.218	1.077	−0.128	0.350	0.720	0.124	0.415	0.623
	**HJ-1B-CCD**	−0.176	1.011	−0.074	0.322	0.827	0.056	0.351	0.786
	**Landsat5-TM**	−0.120	1.036	−0.080	0.275	0.804	0.035	0.390	0.751
	**ALOS-AVNIR-2**	−0.359	1.149	−0.178	0.123	0.692	0.108	0.421	0.643
	**RV (%)**	7.01	4.46	3.81	7.24	5.49	3.77	2.37	6.81
Method of 0.1% index scaling	**Landsat7-ETM+**	0.286	0.757	0.156	0.508	0.572	0.143	0.506	0.526
	**HJ-1B-CCD**	0.323	0.749	0.161	0.504	0.647	0.101	0.465	0.619
	**Landsat5-TM**	0.301	0.752	0.154	0.459	0.632	0.128	0.484	0.583
	**ALOS-AVNIR-2**	0.310	0.756	0.166	0.442	0.558	0.145	0.525	0.516
	**RV (%)**	1.14	0.28	0.46	2.77	3.71	1.48	2.05	3.96

**Table 4.** The performance (classification accuracy, CA) of classification tree models developed for 2009 images normalized using Method of 0.1% index scaling when applied to ETM+ image pairs from 2010 (13 March and 21 September). Values are number of field observations.

**Actual**	**Predicted**				**CA (%)**	**Omission Error**

	**Emergent Vegetation**	**Floating-Leaf Vegetation**	**Submerged Vegetation**	**Other Types**		
**Emergent vegetation**	72	7	0	0	85.7	8.86
**Floating-leaf vegetation**	5	130	6	2	83.9	9.09
**Submerged vegetation**	0	5	96	8	78.0	11.93
**Other types**	0	0	8	173	90.6	4.42
**Commission error**	6.49	8.45	12.73	5.46		

**Table 5.** The performance (classification accuracy, CA) of classification tree models developed for 2009 images normalized using Method of 0.1% index scaling when applied to CCD image pairs from 2010 (10 March and 21 September). Values are number of field observations.

**Actual**	**Predicted**				**CA (%)**	**Omission Error**

	**Emergent Vegetation**	**Floating-Leaf Vegetation**	**Submerged Vegetation**	**Other Types**		
**Emergent vegetation**	75	4	0	1	90.4	6.25
**Floating-leaf vegetation**	4	132	5	2	85.2	7.69
**Submerged vegetation**	0	8	96	5	80.7	11.93
**Other types**	0	1	5	168	93.3	3.45
**Commission error**	5.06	8.97	9.43	4.55		
